# Cardio2share: a web-based database of human, animal and cell resources to share and facilitate collaboration in multi-omics based translational cardiovascular research

**DOI:** 10.1093/database/baag069

**Published:** 2026-11-14

**Authors:** Kanita Karaduzovic-Hadziabdic, Dimitris Kardassis, Osman Gursoy, Márton Makai, Tijana Mitic, Monika Stoll, Mehmet Birhan Yilmaz, Miron Sopic, Paolo Magni, Péter Ferdinandy, Bence Ágg, Yvan Devaux

**Affiliations:** Faculty of Engineering and Natural Sciences, International University of Sarajevo, Sarajevo/Ilidža 71210, Bosnia and Herzegovina; Laboratory of Biochemistry, University of Crete Medical School and Institute of Molecular Biology and Biotechnology, Foundation for Research and Technology of Hellas, Heraklion GR-70013, Greece; Faculty of Engineering and Natural Sciences, International University of Sarajevo, Sarajevo/Ilidža 71210, Bosnia and Herzegovina; Pediatric Center, MTA Center of Excellence, Semmelweis University, Budapest 1083, Hungary; Department of Pharmacology and Pharmacotherapy, Semmelweis University, Budapest 1089, Hungary; Center for Pharmacology and Drug Research & Development, Semmelweis University, Budapest 1089, Hungary; Centre for Cardiovascular Science, Institute for Neuroscience and Cardiovascular Research, University of Edinburgh, Edinburgh EH16 4TJ, Scotland, UK; Institute of Human Genetics, Genetic Epidemiology, University of Münster, Münster 48149, Germany; Department of Cardiology, Dokuz Eylül University Faculty of Medicine, İzmir 35340, Turkey; Department of Medical Biochemistry, Faculty of Pharmacy, University of Belgrade, Belgrade 11221, Serbia; Cardiovascular Research Unit, Department of Precision Health, Luxembourg Institute of Health, Strassen L-1445, Luxembourg; Department of Pharmacological and Biomolecular Sciences ‘Rodolfo Paoletti’, Università degli Studi di Milano, Milan 20133, Italy; IRCCS MultiMedica, Milan 20138, Italy; Department of Pharmacology and Pharmacotherapy, Semmelweis University, Budapest 1089, Hungary; Center for Pharmacology and Drug Research & Development, Semmelweis University, Budapest 1089, Hungary; Pharmahungary Group, Szeged 6722, Hungary; Department of Pharmacology and Pharmacotherapy, Semmelweis University, Budapest 1089, Hungary; Center for Pharmacology and Drug Research & Development, Semmelweis University, Budapest 1089, Hungary; Pharmahungary Group, Szeged 6722, Hungary; Cardiovascular Research Unit, Department of Precision Health, Luxembourg Institute of Health, Strassen L-1445, Luxembourg

## Abstract

Complex and multifactorial diseases such as cardiovascular diseases are characterized by dysregulation of multiple biological pathways. To tackle this complexity, collaborative work between international experts in different fields, with complementary resources, is needed. However, finding the right partner with the right expertise or resources can be challenging. Interdisciplinary COST Action networks CardioRNA and AtheroNET have joined forces to address the need for an easily accessible inventory of resources to catalyze collaborative cardiovascular research. Their resulting platform, named Cardio2share (https://cardio2share.com), is a freely accessible web-based database containing metadata from human cohorts and clinical datasets, animal and cell models available for sharing. Cardio2share contains the curated metadata of patient cohorts, animal and cell models associated with their owner/provider to be contacted for sharing. The database is particularly useful for research projects integrating different omic layers, for the discovery and development of new biomarkers requiring validation in several independent patient cohorts, or for translational projects involving both in vivo and in vitro experiments. By stimulating collaborative efforts, data exchange and reuse in line with the FAIR principles and the 3R policy for animal experimentation, the Cardio2share database will facilitate collaboration between academia, hospitals and the industry, hence improving the translation of results from basic research to clinical application.

## Graphical Abstract

**Graphical Abstract:**
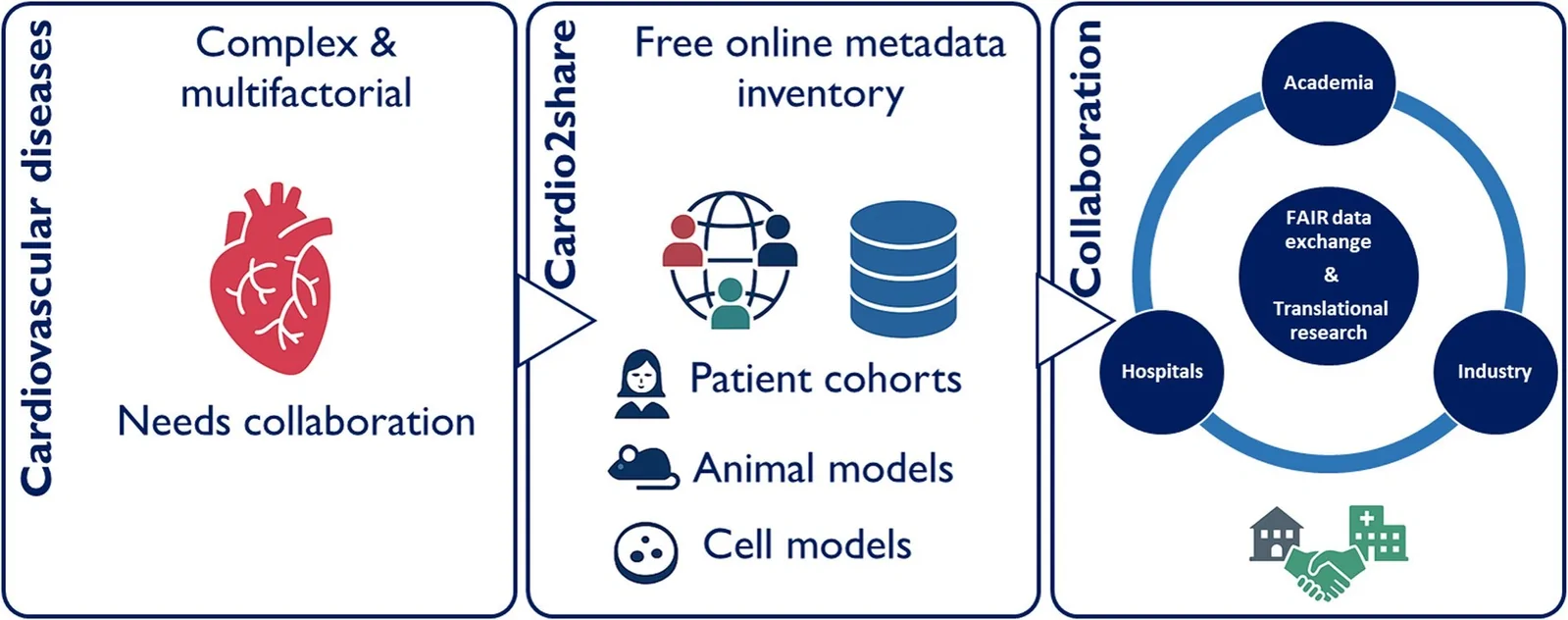

## Introduction

Multiple biological pathways are dysregulated in complex and multifactorial diseases such as cancer, neurological disorders, and cardiovascular disease. Collaboration amongst international experts across multiple countries and fields is necessary to address this complexity [1]. Among available networking opportunities, the European Commission funds the COST Association (European Cooperation in Science and Technology), which fosters the development of networks of researchers throughout Europe and in other partner countries. The COST Action CardioRNA [2] and the COST Action AtheroNET [3] are two examples of such networks, aiming to catalyze collaborative research within the field of cardiovascular disease [4, 5].

Using high-throughput omic techniques such as genomics, transcriptomics, proteomics, and metabolomics to gain a comprehensive and holistic view of biological systems is a prominent and necessary trend in cardiovascular research [6]. Through the concept of unbiased target identification, researchers aim to avoid the biases of the single-target approach, which often overlooks crucial interacting mediators. By integrating bioinformatics and network theory tools, researchers can objectively analyze complex datasets to identify potential targets for therapeutic interventions. Network theory allows for the mathematical modeling [7, 8] and visualization [9, 10] of molecular interactions, and facilitates the discovery of key mechanisms leading to the development of diseases or adverse effects of drugs [11–14]. However, such analyses require synergistic know-how, and a high level of validation.

For example, when searching for novel biomarkers, extensive validation across multiple patient cohorts is essential to ensure the robustness of findings. In the preclinical development of novel drugs, several cellular and animal models are needed to address safety, specificity and effectiveness. This explains why research activities have become highly interdisciplinary. When planning a novel research project, investigators have to build a consortium gathering the necessary and complementary expertise. However, finding the right partners with the right expertise, or the right patient cohorts, or the right animal or cellular models, can be challenging, especially for early-career investigators. To cope with this challenge, and to facilitate samples and data sharing in line with the FAIR principles [15] and the 3R policy for animal experimentation, members of the COST Actions CardioRNA and AtheroNET have designed and implemented an online database, named Cardio2share (https://cardio2share.com). Cardio2share is a freely accessible web-based database that helps researchers to find real-world clinical validation datasets or suitable animal or cellular models available for sharing to facilitate preclinical studies in the complex and multifactorial field of cardiovascular disease (Graphical abstract).

## Methods

Fig. 1 shows the database scheme, illustrated by a schematic overview of the data tables and an entity relationship diagram (Supplementary Fig. 1). It depicts the database structure and forms the back bone of the system developed with Drupal [16] and running on PHP [17]. PHP is a widely used open source scripting language especially suited for web development. Drupal is a free and open-source content management system, known for its flexibility and robust framework for building complex websites and applications. Drupal offers a modular architecture that allows developers to create custom modules tailored to specific functionalities required by the website. Additionally, it supports a wide range of third-party integrations, which were utilized to extend the site’s capabilities. Deployment and maintenance of the website leveraged various Drupal modules (150 modules), including key modules such as Views, CTools, and Pathauto, which streamline content management, enhance user experience, and optimize website navigation.

**Figure 1:**
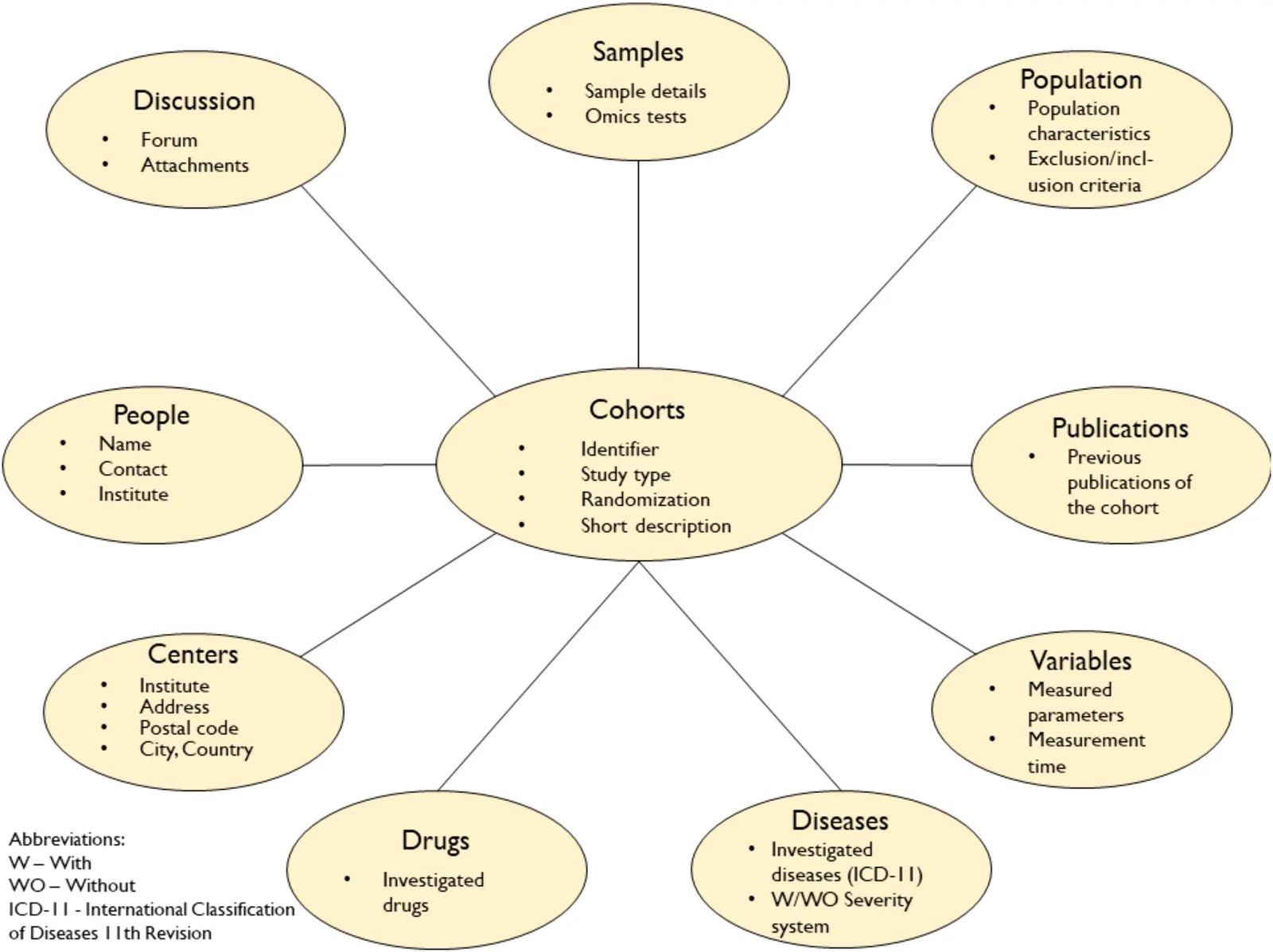
Schematic overview of the data tables in the Cardio2share database.

Cardio2share is available at https://cardio2share.com. Cardio2share was developed and implemented at the International University of Sarajevo (IUS), Bosnia and Herzegovina. The database was designed and is hosted by Semmelweis University, Budapest, Hungary, using a GNU/Linux based server that complies with current industrial standards. The database stores metadata summarizing major characteristics of uploaded models, including cohorts, animal and cell models. While actual data related to these models are not part of the database scheme, researchers can specify external links in the database pointing to publicly available resources related to their model, such as an official website, publications and deposited omics data.

The patient cohort metadata that can be uploaded to the database by registered users are the following:

- *Basic details of the cohort*: name, unique identifier, cohort URL, official URL, start and completion dates, randomization, openness for collaboration, study type, principal investigator, contact person and their contact details, and a short description about the cohort
- *Cohort institutes*: list of institutions that contributed to the cohort with name of the institute, address, postal code, city and country for each
- *People:* list of researchers and coordinators; name, title, profession, contact details and institute
- *Drugs administered/studied in the cohort*: name and code according to the Anatomical Therapeutic Chemical (ATC) classification system
- *Diseases and co-morbidities investigated in the cohort*: name, severity system and stage, if there is any
- *Variables*: name, time of measurement expressed in number of days (e.g. clinical parameters, such as height or weight, cardiovascular risk factors like smoking or hypertension and various echocardiographic parameters)
- *Samples*: type, quantity, associated diseases, omics tests performed and whether the samples are available for sharing.
- *Omics datasets*: type (e.g. RNA sequencing, DNA microarray), accessibility in external repositories (e.g. Gene Expression Omnibus—GEO [18], ArrayExpress [19] or Sequence Read Archive—SRA [20]) by specifying accession number, repository, URL and shareability.
- *Publications related to the cohort*: title, authors, journal, publication date, full text URL and PubMed ID
- *Population related to the cohort*: name, size, sex distribution, age range (minimum and maximum), and inclusion and exclusion criteria used in the study.
- *Discussion*: a platform for easy communication between the researchers and the database administrator

To improve interoperability, standardized databases were used for the classification of drugs and diseases, namely the ATC classification system [21] and the International Classification of Diseases 11th Revision (ICD-11) [22]. A drug or more generally a group of drugs administered in the cohort can be specified by ATC names and codes at all five levels of the ATC classification system. Diseases belonging to chapter 11–‘Diseases of the circulatory system’ of the ICD-11 classification system have been imported into the database, including ‘additional foundation children nodes’, i.e. diseases that primarily fall into a different chapter, but can also be secondarily classified into chapter 11. For example, LA90.40 ‘Congenital renal artery stenosis’ primarily belongs to chapter 20–‘Developmental anomalies’, but secondarily affects the circulatory system. Initial values for additional categories like severity systems and variables were chosen based on the recommendation of experts participating in Working Group 3 of CardioRNA COST Action (CA17129), and can be further extended based on suggestions by the users of the database. For example, for heart failure the NYHA disease severity system [23] is already available in the database, and also a set of frequently studied clinical and laboratory parameters can be specified for each cohort.

The animal model metadata that can be entered to the database by registered users are the following:

- *Basic details of the animal model*: name, unique identifier, inventory URL, official URL, date that the model was generated, model background, openness for collaboration, study type, principal investigator, contact person and their contact details, species, genetic background, genetic modification, method of modification, cre recombinase, zygosity, disease model, model availability, repository and a short description about the animal model.
- *Animal model institutions*: list of institutions that contributed to the animal model with name of the institute, address, postal code, city and country for each.
- *List of researchers and coordinators*: name, title, profession, contact details and institute
- *Publications related to the animal model*: title, authors, journal, publication date, full text URL and PubMed ID
- *Discussion*: a platform for easy communication between the researchers and the database administrator

The cell model metadata that can be uploaded to the database by registered users are the following:

- *Basic details of the cell model*: name, unique identifier, inventory URL, official URL, openness for collaboration, principal investigator, contact person and their contact details, species, tissue of origin, cell line/primary cells, model type, disease model, availability, data type, repository and a short description about the cell model.
- *Cell model institutions*: list of institutions that contributed to the cell model with name of the institute, address, postal code, city and country for each.
- *List of researchers and coordinators*: name, title, profession, contact details and institute
- *Publications related to the cell model*: title, authors, journal, publication date, full text URL and PubMed ID
- *Discussion*: a platform for easy communication between the researchers and the database administrator

Before publication on the public interface, newly submitted or modified entries undergo manual review by the database administrator, who is a medical professional. The database administrator assesses the submitted information for completeness, validity, consistency, compliance with the database standards and FAIR principles, and conformity with the Privacy Policy. Validity checks include, where applicable, verification that provided links and email addresses are functional, while consistency is assessed by comparing the submitted information with corresponding publicly available sources, such as the relevant study website. The public accessibility of the referenced online resources is also verified where applicable. Only entries that successfully undergo this review are approved and made publicly visible in Cardio2share.

In accordance with current data protection directives, the need to store personal data will be reviewed every five years from the launch of the database, and on personal request, the published personal data can be withdrawn at any time.

## Results

### User types

The web application has three main types of users:

1. Public user: can freely search for the available cohorts and animal or cell models in the database, without the need for registration.
2. Registered user: can search for and enter cohort, animal or cell model information into the database.
3. Database administrator: responsible for database maintenance, verification and approval of the data entered by registered users (Fig. 2).

**Figure 2:**
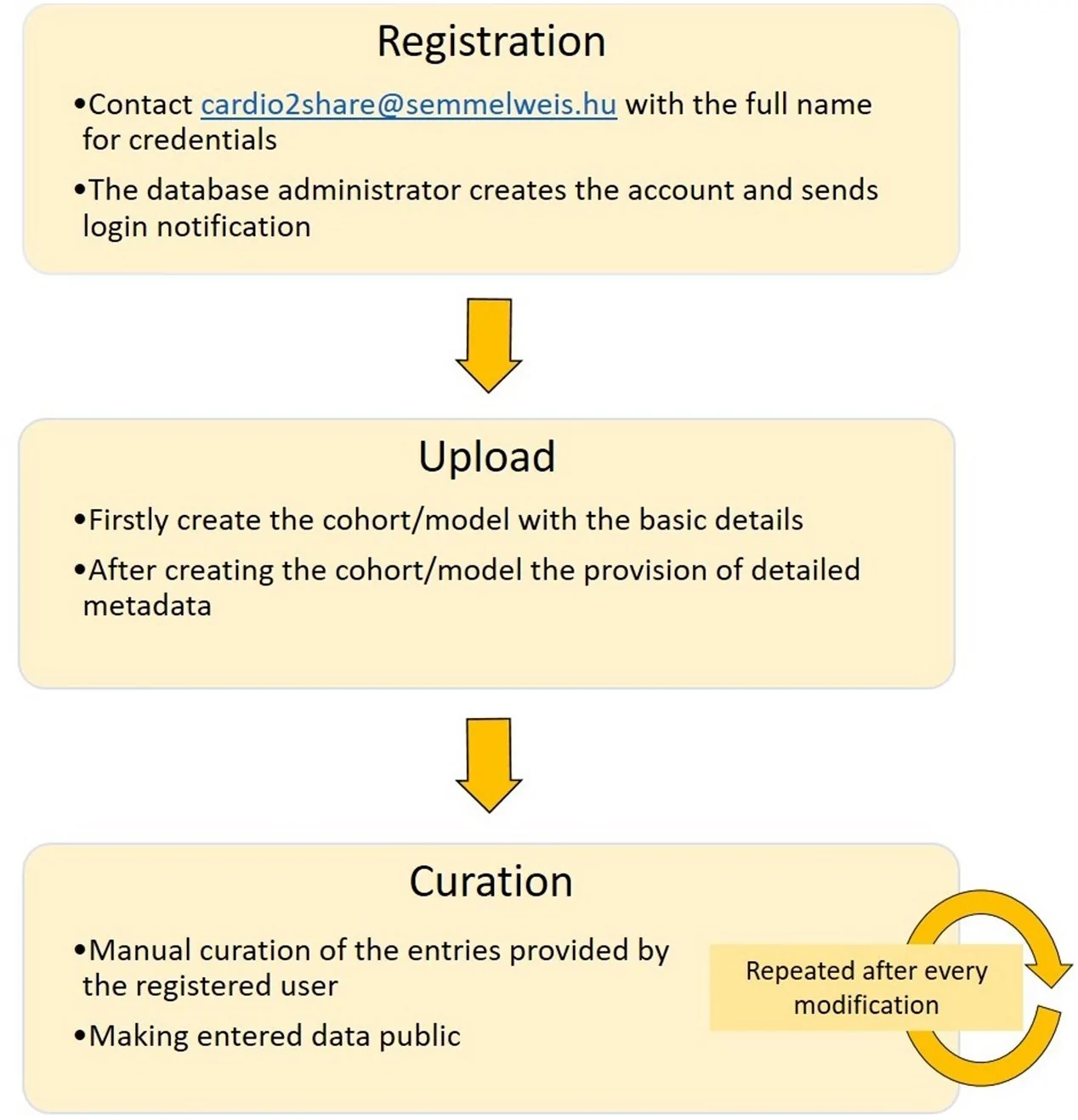
Schematic overview of the operation of the database.

### Public user interface

The home page of the website contains information on the registration process and introduces the CardioRNA and AtheroNET COST Actions (Fig. 3). By selecting ‘Cohorts’ from the main menu bar users are directed to the *Available Cohorts* page (Fig. 4a), which displays a list of all the existing cohorts entered by registered users and approved by the database administrator. The page also includes the database’s search function, allowing users to filter the database content by cohort name, diseases or variables.

**Figure 3:**
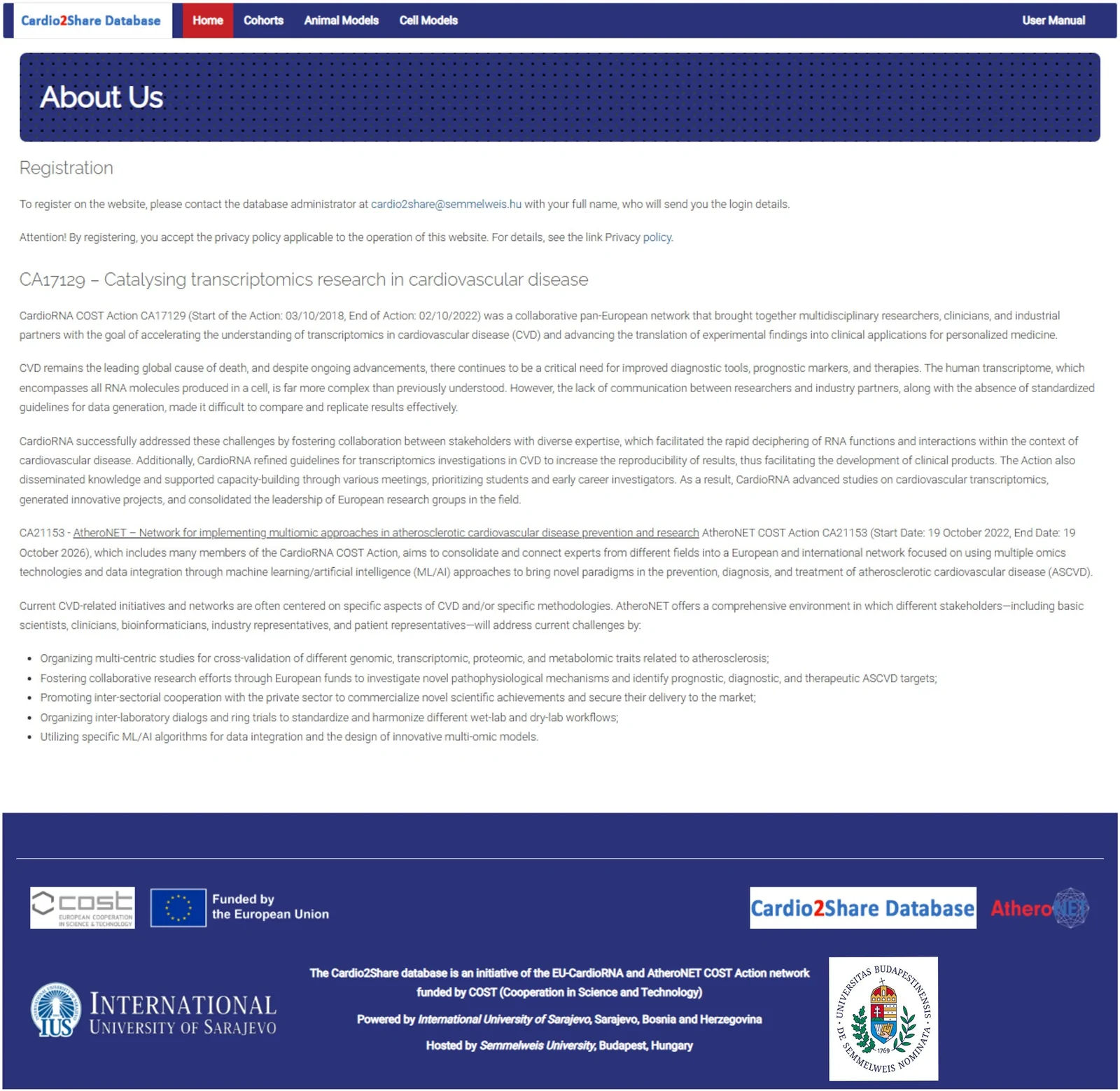
Screenshot of the home page of the Cardio2share database with instructions on the registration process.

**Figure 4:**
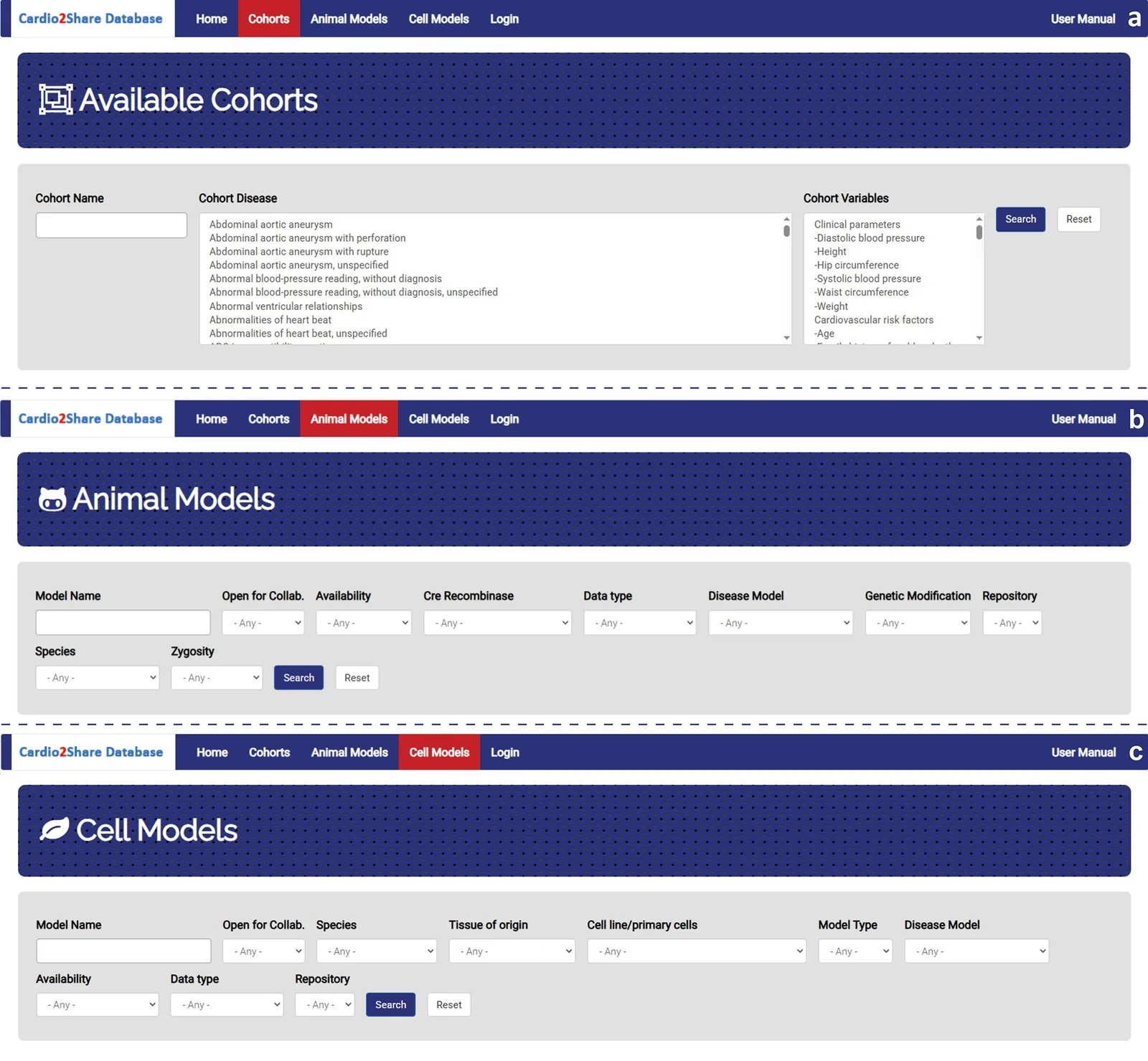
Screenshot of the *(a) Cohorts, (b) Animal* and (c) *Cell models* page, where uploaded cohorts and experimental models are listed.

Similarly, selecting ‘Animal models‘, or ‘Cell models‘ from the main menu bar, directs users to the corresponding Animal Models (Fig. 4b) or Cell Models (Fig. 4c) pages, which list all approved animal or cell model inventories entered by registered users. As with the Available Cohorts page, these pages also include a search function, allowing users to filter database content by various attributes (model name, openness for collaboration, availability, etc.).

A link to a User Manual explaining the functioning of the database is provided at the upper right corner of each page of the website.

### Registered user interface

While the database is freely available for consulting, registration is necessary for entering one or several cohorts, animal models or cell models. To this end, users send their full name to the database administrator at cardio2share@semmelweis.hu. Login credentials (username and password) are then provided as a reply. Using these credentials, users can log into the database and create new cohorts or models. After creating a cohort, animal or cell model, users may enter metadata for cohorts, animal or cell models as detailed in the Methods section. Subsequently, all the data entered into the database undergoes manual verification and validation by the database administrator to ensure correctness and consistency. The data will not be made publicly available until the verification step is completed. Any modifications to cohort or model data will result in the verification procedure to be repeated (Fig. 2).

### Creation of new cohort, animal and cell model

After logging in, the user can create new cohorts, models, institutions or persons (principal investigator or contact person) by selecting the ‘New’ option from the main menu bar.

Fig. 5 shows the *Create Cohort* page which includes a form enabling users to input the cohort’s basic characteristics (name, unique identifier, cohort url, etc.), as described in the *Methods* section. After a new cohort has been created, multiple tabs (Institution, People, Drugs, Diseases, Variables, Publications, Population, Samples, Discussion) allow users to enter additional cohort details in line with the database scheme described in Fig. 1. People tab contains a list of all individuals, from which users can select a principal investigator and/or contact person via a dropdown menu when creating a cohort on the *Create Cohort* page. This list is shared across cohorts, animal and cell model inventories. Furthermore, when a new cohort, animal or cell model is added, a privacy icon is shown indicating that the newly added cohort, animal or cell model will not be displayed in the public view until it has been reviewed and approved by the database administrator, thereby ensuring the accuracy and reliability of the database.

**Figure 5:**
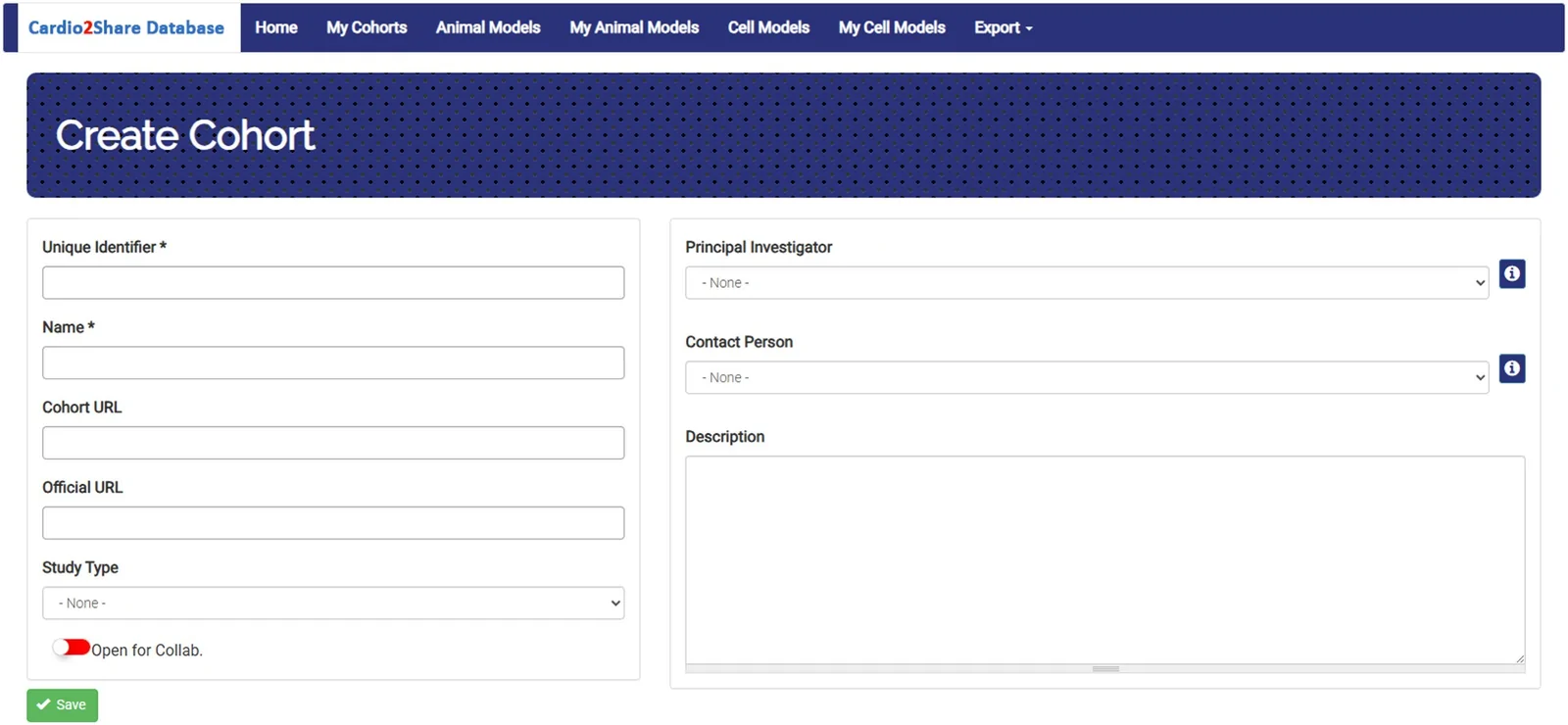
Screenshot of the New → *Create Cohort* page where basic characteristics of a cohort can be specified.

The *My Cohorts* option directs registered users to the page listing only cohorts they have created.

Features of each tab for patient cohorts are described as follows:

- Institution: Includes all the institutions associated with the cohort. For the current cohort, cohort institution can be added either by creating a new institution by clicking the ‘New Institution’ button, or users can select an existing institution from the list of institutions by selecting the ‘Select from List’ button.
- People: Contains a list of all people entered by the registered users. When creating a cohort (in Create Cohorts page), users can select a Principal Investigator or a Contact Person from this list via a dropdown menu. New persons can be added via the ‘New Person’ button.
- Drugs: Contains drugs associated with the created cohort. New cohort drug can be added via the New Cohort Drug button, or users can select an existing drug from the list of drugs according to the ATC classification system by selecting the ‘Select from List’ button.
- Diseases: Contains diseases associated with the created cohort. New cohort disease can be added by selecting the ‘New Cohort Disease’ button, allowing users to select a cohort disease name from ICD-11 disease severity system and disease stage.
- Variables: Contains a list of all the variables associated with the created cohort. New cohort variables can be added by selecting the ‘New Cohort Variable’ button, allowing users to select from a list of predefined options, such as clinical parameters, cardiovascular risk factors, ECG, coronary angiogram, echocardiographic parameters, symptoms and blood markers from a dropdown menu. It also allows users to specify the duration (in days) for which the selected variable was measured.
- Publications: Contains a list of publications associated with the cohort (title, authors, journal, publication date, URL, and PubMed ID)
- Population: Contains a list of cohort populations including population details such as name, size, sex distribution, age range (minimum and maximum), and inclusion and exclusion criteria used in the study. New cohort population can be added by selecting the ‘New Cohort Population’ button.
- Samples: Contains available cohort samples, associated sample diseases, omics tests performed, whether the samples are available for sharing, and associated omics datasets (type), accessibility in external repositories (e.g. Gene Expression Omnibus—GEO, ArrayExpress or Sequence Read Archive—SRA) by specifying accession number, repository, URL and shareability.
- Discussion: a discussion tab for easy communication between the researchers and the database administrator.

For animal and cell models, the database pages include forms that allow users to input animal or cell model information. The main page of animal and cell models includes Institutions, People, Publications and Discussion tabs allowing users to input additional animal or cell model details. The following information is available for animal models: Name, Open for collaboration, Availability, Cre recombinase, Data type, Disease model, Genetic modification, Repository, Species, Zygosity. The following information is available for cell models: Name, Open for collaboration, Species, Tissue of origin, Cell line/primary cells, Model type, Disease model, Availability, Data type, Repository. Registered users can also export all animal and cell model inventories to an Excel file by selecting Export → All Animal Models or Export → All Cell Models from the main menu bar. By selecting My Animal Models or My Cell Models, registered users will be directed to the page listing only animal or cell models they have created.

### Database administrator interface and roles

The database administrator of Cardio2share is responsible for overseeing and maintaining the database. Their primary tasks include:

- Database maintenance—Ensure the database runs smoothly, manage updates, resolve technical issues, and monitor overall system integrity.
- Creation and management of user accounts—Generate login credentials for new registered users, and manage access permissions.
- Verification and approval of new data—Review all data entered by registered users (cohorts, animal models, and cell models) to ensure accuracy, consistency, and compliance with database standards. Only approved entries become visible on the public interface.

The key features and workflow for the verification and approval of new data include:

*Entry overview*

Each pending entry is displayed in a table with columns for:

- Entry type—Specifies whether the item is a cohort, cohort publication, cohort variable, or other database content.

- Entry name—Name or unique identifier of the submission.

- Created on/Last modified date—Timestamps for when the entry was first created and last updated.

- Author information—Name and email of the registered user who submitted the entry.

*Action options*

For each pending item, the database administrator can:

- View entry details—Opens the full submission for inspection.

- Edit—Modify the entry if corrections are needed.

- Delete—Remove incorrect or inappropriate entries.

- Confirm—Approve the entry, making it visible in the public database.

- Unconfirm—Revert a previously confirmed entry to pending status if updates or corrections are required.

*Search and filter*

Database administrator can filter entries by entry type, cohort name, or confirmation status (whether the entry is confirmed or not), making it easier to prioritize verification tasks.

The approval workflow is as follows:

1. A registered user submits a new cohort, animal, or cell model entry.
2. The entry appears in the Pending approval/Confirmations Table in the Database administrator view.
3. The database administrator reviews the entry details for accuracy, completeness, and compliance with database standards.
4. The database administrator either confirms, edits, or deletes the entry.
5. Once confirmed, the entry becomes publicly visible, ensuring that only verified data is accessible to general users.

The database administrator interface ensures quality control and consistency across the database while allowing the database administrator to manage submissions efficiently.

### Case study

A concrete example of Cardio2share database use is presented hereby. With their in-house cohort of patients with acute myocardial infarction, an investigator discovered a potential biomarker of the development of heart failure in whole blood samples. Willing to independently validate this finding, the investigator interrogated the Cardio2share database for cohorts of acute myocardial infarction patients and found the LUCKY cohort with whole blood samples available for sharing. They contacted the principal investigator of this cohort and started the collaboration and independent validation. Willing to address in vitro functional relationships between the new biomarker and disease progression, the investigator further searched the Cardio2share database and found an endothelial cell line available for sharing to test the role of the biomarker in post-acute myocardial infarction angiogenesis and remodeling. Lastly, the investigator decided to conduct in vivo experiments and found in Cardio2share database a collaborator performing coronary ligation in mice as a model of acute myocardial infarction. Overall, Cardio2share use allowed this investigator to establish a tri-partite partnership to validate the newly discovered biomarker.

## Discussion

In cardiovascular research, valuable datasets from clinical trials, imaging studies, omics research and experiments in pre-clinical model systems often remain out of reach due to limited data-sharing practices and a general lack of awareness about the existence of these datasets or associated human-sample biobanks.

The main goal of Cardio2share is to implement a database of patient cohorts and experimental models of cardiovascular disease available for sharing and re-use, in line with the FAIR principles and the 3R policy for animal experimentation. The database provides a collection of key characteristics of cohorts, animal and cell models and contact details of principal investigators and the cohort’s contact person. Cardio2share was created in order to promote the dual general requirement of ensuring the reproducibility of findings from biomarker studies through the access and use of multiple patient cohorts available for validation purposes, and sharing available cell and animal models to avoid duplication of work and benefit from each other’s expertise. Overall, Cardio2share aims to facilitate and accelerate collaborations among key stakeholders in cardiovascular research, including clinicians, scientists, and industry partners [24, 25]. Shared access to the data collected in Cardio2share, in line with the general aims of COST actions, will promote knowledge exchange, dissemination of best practices, and efficient transfer of resources, ultimately enhancing collective learning, innovation, and long-term contributions across relevant fields. Moreover, Cardio2share may support strategic prioritization of future research by revealing areas where key population cohorts or experimental models are still lacking.

While the current holdings of the database are relatively limited, a dissemination campaign including news on social and personal networks, presentations during meetings, contents in newsletters of personal networks (e.g. European Society of Cardiology Working Groups) will inform the research community about the existence and benefits of the database. Cohorts of patients and preclinical models will be subsequently entered into the database, stimulating collaborative work.

By encouraging teamwork, data sharing, and data reuse, Cardio2share is expected to accelerate the translation of research results into clinical application ultimately benefiting patients and healthcare systems.

## Supplementary Material

baag069_Supplemental_Files
